# Suppressing Non-Specific Binding of Proteins onto Electrode Surfaces in the Development of Electrochemical Immunosensors

**DOI:** 10.3390/bios9010015

**Published:** 2019-01-18

**Authors:** Jesús E. Contreras-Naranjo, Oscar Aguilar

**Affiliations:** Tecnologico de Monterrey, Escuela de Ingeniería y Ciencias. Ave. Eugenio Garza Sada 2501, Monterrey 64849, N.L., Mexico; a00825447@itesm.mx

**Keywords:** electrochemical immunosensors (EIs), EI sensitivity enhancement, non-specific binding (NSB), protein adsorption

## Abstract

Electrochemical immunosensors, EIs, are systems that combine the analytical power of electrochemical techniques and the high selectivity and specificity of antibodies in a solid phase immunoassay for target analyte. In EIs, the most used transducer platforms are screen printed electrodes, SPEs. Some characteristics of EIs are their low cost, portability for point of care testing (POCT) applications, high specificity and selectivity to the target molecule, low sample and reagent consumption and easy to use. Despite all these attractive features, still exist one to cover and it is the enhancement of the sensitivity of the EIs. In this review, an approach to understand how this can be achieved is presented. First, it is necessary to comprise thoroughly all the complex phenomena that happen simultaneously in the protein-surface interface when adsorption of the protein occurs. Physicochemical properties of the protein and the surface as well as the adsorption phenomena influence the sensitivity of the EIs. From this point, some strategies to suppress non-specific binding, NSB, of proteins onto electrode surfaces in order to improve the sensitivity of EIs are mentioned.

## 1. Introduction

The International Union of Pure and Applied Chemistry (IUPAC) defined in 2011 an electrochemical immunosensor (EI), as an integrated analytical device where the biorecognition event is based on an antigen/antibody reaction, which can transduce the product molecules of this reaction into an electric signal through the electrode surface (transducer) and quantify the amount of antigen present in the sample [1]. EIs can be applied in several areas of the knowledge and industry. In the last decade, the interest in building new sensor platforms that advance into commercial sensing devices for early clinical diagnosis of cancer and heart diseases has increased [2]. So, in this sense, a wide variety of studies about EIs have been carried out, mainly focusing on the improvement of the analytical efficiency of the device. In other words, the goal in the design and construction of sensing platforms is to measure the smallest possible amount of the target analyte present in a real complex sample with high precision and accuracy [3]. To achieve this goal, it is necessary to create simple and innovative sensor platforms able to suppress non-specific binding (NSB) of undesirable proteins or molecules in the electrode surface [4].

Various strategies have been reported for minimizing NSB and are classified in two groups: physical and chemical surface modifications. Physical modification strategies are performed attaching molecules directly to the surface, e.g., blocking buffer solutions, or by forming a complex with other particles, e.g., avidin coated surfaces. Such physical protein adsorption is governed by van der Waals forces, hydrophobic interactions, electrostatic interactions and hydrogen bonding. On the other hand, chemical modification strategies are more specific than physical adsorption and include chemical reaction between different residues (e.g., amine, thiol, carboxyl) of interacting molecules. Chemical modification strategies include: i) allotropic modification of carbon, e.g., carbon nanostructures, ii) modification by metal nanoparticles, e.g., metallic Au/Ag nanoparticles and magnetic beads, iii) polymerization, e.g., polyethylene glycol (PEG), oligo (ethylene glycol) (oEG) and conducting polymers, iv) self-assembled monolayers (SAMs), v) diazonium salt surface chemistry and vi) sol-gel chemistry modification [5,6]. 

The present review is focused on a brief description of some basic characteristics of the EIs, types, properties and operation principle of state-of-the-art immunosensors, including the sandwich enzyme-linked immunosorbent assay (ELISA) coupled with electrochemical detection. A critical description of the main issues regarding the assembly of screen printed electrodes (SPEs), which are the electrode surfaces most commonly used in the design of this type of platforms, is presented. This includes a description of the physical and chemical properties of the protein and the surface which influence the behavior of the protein adsorption process and help in the control of the specific adsorption and orientation of proteins on any electrode surface to improve the sensitivity of the EIs. Finally, a brief description of the physical and chemical surface modification strategies developed to suppress NSB of antibodies and proteins onto electrode surfaces is presented.

## 2. Electrochemical Immunosensors, EIs

**Figure 1:**
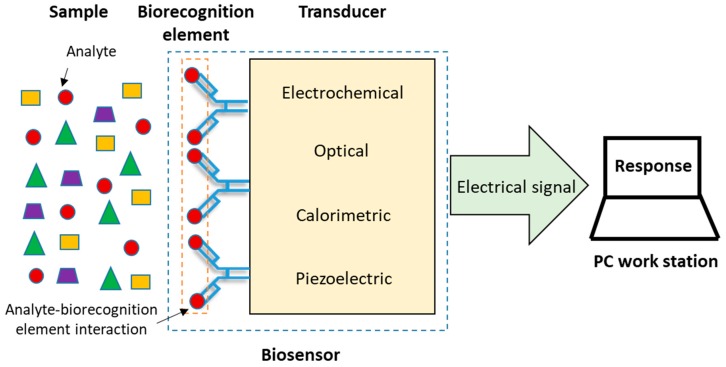
Components of a biosensor [2].

According to the IUPAC, a biosensor is an analytical device that incorporates a biological element immobilized on the surface of a physicochemical transducer, which recognizes the target molecule or analyte present in a sample and, by means of the transducer, this event of biorecognition is transduced into a discrete or continuous electric signal, directly proportional to the amount of analyte in the sample [7,8,9,10]. Finally, the electrical signal is shown as a response in a computer work station (Figure 1). In general, biosensors are classified by their biorecognition element or their transduction principle [11,12]. Antibodies, enzymes, cells/tissues, nucleic acids and aptamers are some of the elements of biorecognition frequently used. The principal types of transducers are: optical [13,14], electrochemical [15,16,17], calorimetric [18,19] and piezoelectric [20,21].

The most outstanding characteristics of these devices, which make them highly attractive options as analytical tools, are: their selectivity and specificity, high sensitivity, responsiveness that leads to a short analysis time, their ability to be included in integrated systems, ease of automation, ability to work in real time, its versatility and low cost, among others [22,23]. In the EI, an affinity biosensor, the immobilized biological entities are antibodies (Abs) that recognize the target analyte (antigen, Ag), while an electrochemical transducer converts the binding event between the antigen and antibody, resulting in the formation of the complex antigen-antibody (Ag-Ab), into a useful electrical signal, either an electrical current (amperometric immunosensors), a potential difference (potentiometric immunosensors) or a change in resistivity (conductimetric and impedimetric immunosensors) [24,25]. 

Antibodies, specifically monoclonal antibodies, are a very useful biorecognition element in EIs due to the affinity that they have with respect to the analyte of interest, characterized by their high affinity and specificity, good stability and versatility and low cost compared to other elements of biorecognition such as polyclonal antibodies, enzymes, whole cells, nucleic acids and molecularly imprinted polymers (MIPs) [26,27]. Monoclonal antibodies present some advantages compared to polyclonal antibodies. Among these, we find that monoclonal antibodies are very specific for a single epitope in a multivalent antigen, contrary to what happens in polyclonal antibodies that recognize and bind to the same antigen but can be combined with different epitopes. Another advantage of monoclonal antibodies is that the hybridoma cell line that produces them is potentially "immortal" and can produce the same antibodies in a constant and indefinite manner [28,29].

EIs combine the high sensitivity of electrochemical techniques and the high selectivity and specificity of antibodies to the target analyte. The most common type of amperometric immunosensors is the ELISA sandwich immunoassay with electrochemical detection. Usually, a sandwich immuno-complex (^Btn^Ab-Ag-Ab^HRP^) is composed of a biotinylated primary antibody, also called capture antibody (^Btn^Ab), the analyte, also called antigen (Ag) and a second antibody (detection antibody) labelled with a redox enzyme, e.g., horseradish peroxidase, HRP (Ab^HRP^) adsorbed onto an avidin-coated electrode surface due to the high affinity of the avidin-biotin system (or its analogous streptavidin and neutravidin). 

**Figure 2:**
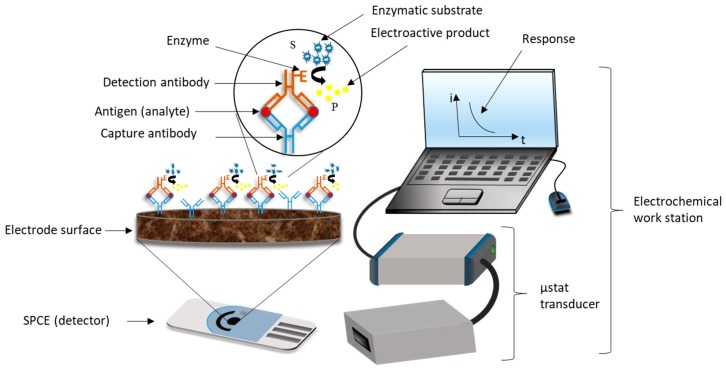
Basic analytical principle of an ELISA sandwich amperometric immunosensor using screen printed carbon electrodes (SPCEs) [32].

The purpose of this test is to quantify the number of antigens (target analyte) present in complex samples such as human serum through the detection of the immuno-complex, via an electron transfer reaction between the redox enzyme, the enzyme substrate (S), e.g., hydrogen peroxide (H_2_O_2_), and the redox mediator, e.g., 3,3′,5,5′-tetramethylbenzidine (TMB). The HRP oxidize the TMB into TMB^+^ in the presence of H_2_O_2_, releasing one electron per mole of TMB oxidized. TMB^+^ is reduced to TMB by the imposition of a potential. Thus, as a consequence of the oxidation and reduction of the electroactive product (P) in the electrode surface, a steady-state current is established in the process (Figure 2). If the ^Btn^Ab-Ag-Ab^HRP^ complex does not form, the measurable current values will be considerably low, which means a negative result. In contrast, high current values indicate a positive result [30,31,32,33]. 

An example of an electrochemical ELISA sandwich immunoassay was proposed by Zhang et al. in 2015 for the quantification of prostate-specific antigen (PSA) using redox and catalysis “all-in-one” infinite coordination polymer (PtNP@ICP) as a signal tag for the label of PSA on the polyamidoamine dendrimers-modified glassy carbon electrode interface. Their results showed a limit of detection (LOD) of 0.3 pg/mL at 3s_B_ [34].

As it is known, EIs are based on the antibody-antigen interaction and possess high specificity and selectivity for the analyte of interest. Since the antibody is immobilized on small electrode surfaces, this type of biosensors can be miniaturized and easily converted into portable devices with the help of microelectrodes (where the immunological reaction will be carried out), among which SPEs are of great importance [29,30]. Immunosensors have a wide variety of applications (e.g., biomedical, clinical diagnosis, environmental and food industry) compared to enzyme-based sensors, given that antibodies are universal in contrast to enzymes, which depend on each substrate [35]. 

The advantages that EIs present over immunoassays, specifically in relation to the commercial sandwich ELISA test, are their good portability (easy miniaturization) in order to carry out clinical diagnosis in the place where the patient is located (point of care testing, POCT) with a minimum of training required, good sensitivity, low cost, rapid analysis, instrumental simplicity and precise measurements, while maintaining the optical approximation of the ELISA test. On the other hand, adaptation of the ELISA assay to an electrochemical approach is not an easy task, because many factors can affect the signal response and hence the performance analysis regarding sensitivity. A poor signal response may be due to the denaturation or loss of affinity and specificity of antibodies and proteins when they are adsorbed onto the electrode surface, an incorrect orientation of antibodies in its adsorbed state which leads to an increase of the steric hindrance, and cross-reactivity from antibodies to other non-specific molecules present in the sample instead of the antigen of interest. Also, in EIs, it is desirable to generate the enzymatic product directly on the electrode surface to favor the electron transfer rate; moreover, direct adsorption of interacting biomolecules may passivate or poison the electrode surface, thus affecting the electrochemical behavior of the sensor. These are the most common concerns in EIs in order to achieve performances comparable or even better than those of the commercial ELISA optical approach [36,37,38,39].

Methodologies oriented to improve the electrochemical detection of biomolecules of interest in the development of more sensitive platforms employ magnetic beads, nanostructures (carbon nanotubes, CNTs, multiwall carbon nanotubes MWCNTs, Quantum dots), and nanoparticle labels [40,41,42,43].

## 3. Screen Printed Electrodes, SPEs

A wide variety of electrodes have been used as a support in the fabrication of immunosensor devices: carbon paste electrodes, glassy carbon electrodes and gold electrodes. Recently, most of the sensor devices have been constructed onto SPEs. Nowadays, the screen printing microfabrication technology is well stablished, offers high-mass production of solid, planar, small size, low cost, disposable, mechanically robust and highly reliable thick film electrodes; allowing the construction of portable, low cost and pocket size devices for on-site diagnosis [44,45]. A detailed description of the fabrication process of SPEs was reported by Li et al. in 2012 [46]. 

In summary, fabrication of SPEs consists of various steps: selection of the screen or mesh which will define the thick layer film, geometry and size of the SPE, selection and preparation of the inks for the reference, counter and working electrode, selection of the substrate, sequential deposition of layers of ink onto the substrate, drying and curing steps between each layer deposition of the ink [47,48,49]. Even though the exact formulation of the inks is according to the manufacturer as copyright information, it is well stablished that the ink is made mainly by synthetic grade graphite, vinyl or epoxy-based polymeric binder and solvents. Graphite is the electrode material, the binder increases adhesion and mechanical strength, and solvents allow to control the ink viscosity [50]. The control of the ink composition is very important, because any minimal change strongly affects the electron transfer process and changes the overall performance of the sensor device [51,52]. 

**Figure 3:**
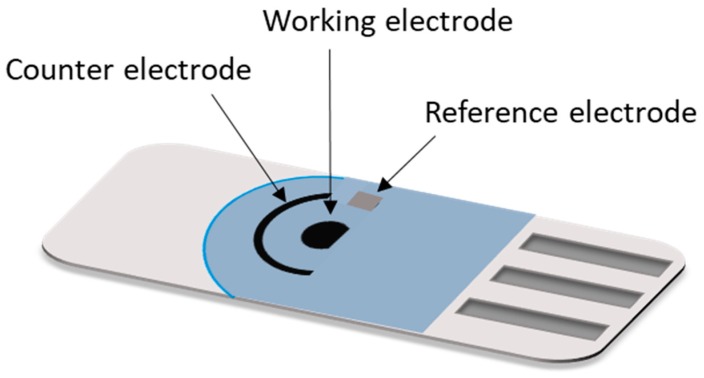
Design of an easy-to-use, disposable and portable SPCE [52,54].

The SPE substrate usually is a non-conducting solid surface material such as alumina, glass, ceramic, plastic, etc. and the electrode conducting parts are made of carbon ink/paste, platinum, gold or other metal pastes. The improvement of the EIs is intimately related to the material of the working electrode on which the reactions of interest occurs. Carbon or other forms of carbon such as graphene, graphite, fullerene, carbon nanotubes, and single/multi-wall carbon nanotubes are most commonly employed as a material in the fabrication of the working electrode. Some characteristics that made carbon suitable for this purpose are its low cost (compare to metals e.g., gold, silver or platinum; which have been also utilized as a working electrode materials and present higher fabrication costs), easiness to modify, chemically inert, good electrical and thermal conductivity, high mechanical and dimensional stability and for electrochemical activities its low background currents and wide potential operational window [53,54,55]. Usually, the material of the reference electrode is Ag/AgCl and the counter electrode generally is made from the same working electrode material [54,55] (Figure 3).

A diversity of SPEs configurations are available nowadays in the market for a broad spectrum of applications which can be personalized according to the customer requirement. Configurations of two electrodes (working and reference electrodes, also called first generation SPEs), three electrodes (working, reference and auxiliary electrodes, also called second generation SPEs) are the most used [56]. In addition, SPEs with multiplex working electrodes, arrays of eight SPEs and of 96 SPEs in a 96-well plate have been developed for the simultaneous detection of multiple biomarkers [57].

Recently, the interest to replace rigid substrates with paper substrates for fabricating SPEs has strongly increased in the development of new electroanalytical tools. Various studies about SPEs based on paper and transparency in order to detect a broad spectrum of biomolecules have been reported [58,59]. Further, wearable, tattoo-based wearable and skin worn electrochemical sensors and biosensors employing SPEs as a sensor platform are a very good alternative to measure in real time physical parameters such as heart rate, respiration rate, oxygenation of the blood, skin temperature, bodily motion, brain activity, and blood pressure, etc., without compromising user comfort. These are some examples of the great versatility of SPEs in the continuous development of bioanalytical devices [60,61].

## 4. Understanding Protein Adsorption on Solid Surfaces to Improve Electrochemical Response

Presently, there is a growing interest to develop new analytical devices that allow high sensitivity, specificity and rapid detection of target biomolecules (proteins, peptides, DNA, RNA, etc.) for early and precise clinical diagnosis. The design of EIs implies the immobilization of antibodies on a monolayer of protein receptors adsorbed on a transducer element, e.g., carbon electrode solid surface [62,63]. 

In order to improve the sensitivity of the EIs, it is necessary to understand the different interactions that occur in the protein-surface interface, in order to optimize protein surface density, proper orientation of the protein, a well-organized compact monolayer, good receptors accessibility, long term stability and minimize non-specific adsorption of the protein. The non-specific adsorption of proteins on the electrode surface is the principal cause of denaturation and loss of binding capacity functions, which results in poor performance of the EIs [64,65]. 

**Figure 4:**
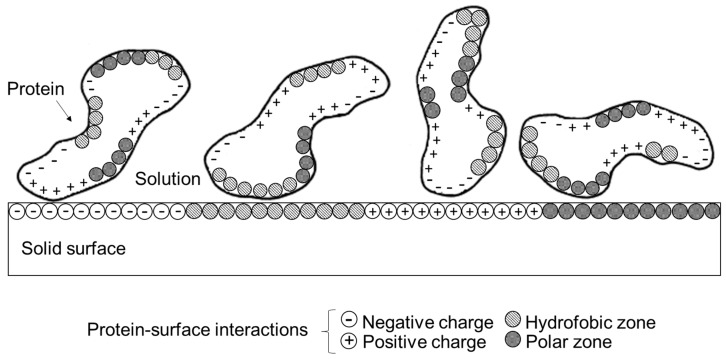
Protein-Surface Interactions [72].

Properties of both the protein and the surface influence the behavior of the adsorption process. The properties of the protein are related to its primary structure, specifically the sequence of amino acids and functional groups available for bonding. The variety of chemical residues cause the protein to interact differently with the groups on the surface [66,67,68] (Figure 4).

The size of the protein also influences how it interacts with the surface. Large protein molecules interact better because they have a greater number of binding sites to contact the surface than in comparison with smaller molecules [69,70]. The charge of the amino acids with electrically charged side chains as well as the distribution of the charge on the surface of the protein and the solid surface can greatly influence the protein-surface interaction and thus the adsorption process [71]. Also, proteins often show a high interaction with the surface near their isoelectric point, pI [72,73]. Bremer et al., in 2004, demonstrated these two points mentioned before, adsorbing IgG molecules on two different hydrophilic surfaces: a negatively charged silica surface and a positively charged amine functionalized silica at different electrolyte ionic strengths and pH values above and below of the isoelectric point, pI of the IgG (pI = 6). They conclude that electrostatic and van der Waals interactions are a combination of the electrostatic interactions between the IgG-surface (hetero-interactions) and lateral interactions between IgG-IgG molecules (homo-interactions) and also that the adsorption rates depend on pH and ionic strength [74].

The properties of the solid surface that influence the adsorption process are similar to those of the protein; such properties can be classified into three groups: geometric, chemical and electrical. Geometric properties have to do with the topography and heterogeneity of the surface. A greater texture (grooves or pores) exposes more surface area of contact with more points of interaction with the protein. In terms of heterogeneity, the non-uniformity of the surface results in domains or regions that may interact differently with the protein [75,76]. 

Chemical composition of the surface determines the availability of functional groups for the interaction with the protein. As an example, Roach et al. in 2005 studied the effects that have functional groups such as methyl, CH_3_ (hydrophobic surface) and hydroxyl, OH (hydrophilic surface) on the adsorption rates and conformation of bovine serum albumin (BSA) and fibrinogen through protein-surface interactions. In the case of BSA, adsorption is carried out via a single step showing a high affinity toward CH_3_ compared to the OH surface functional group. In contrast, fibrinogen, adsorbs faster to both surfaces in a multistage process showing a slightly higher affinity toward the hydrophobic surface [77,78]. 

The potential of the surface influences the structure and composition of the electrolyte solution adjacent to the surface. The combined effects of water molecules, ions and net surface potential will determine whether the interaction with the protein will be improved or impeded [79].

**Figure 5:**
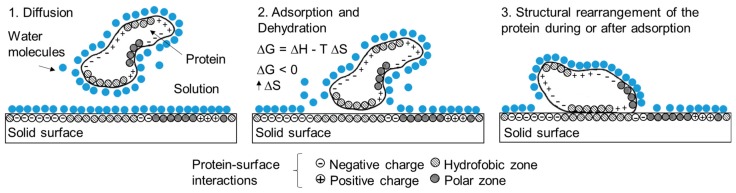
Adsorption process of a protein onto a solid surface [70,72,76].

Adsorption of proteins on electrode surfaces is a very complex process governed by different non-covalent interactions that occur between the protein-surface such as the intermolecular forces of van der Waals, hydrophobic and electrostatic interactions [80]. The adsorption of proteins creates a water-free contact layer where the protein can be unfolding with a distribution of charges in the interface followed by a structural rearrangement, without solvating any of its hydrophobic residues by maximizing the interaction and contact area with the hydrophobic region of the surface (Figure 5). Therefore, adsorption of proteins is an entropically driven process [81,82]. 

Protein molecules change their conformations to a larger extent on hydrophobic surfaces than on hydrophilic surfaces [83]. It has been shown that a strong adsorption of proteins occurs on highly oriented pyrolytic graphite, carbon paste electrode (CPE) or screen printed carbon electrode (SPCE) surfaces. Despite the rearrangement or structural unfolding of the protein, a partial or complete denaturation of the protein does not occur, as it preserves its binding capacity, probably due to an increase in bio-compatibility of the electrode surface [84,85]. In contrast, in bare metal electrodes such as mercury, silver, gold and platinum the unfolding of the protein leads to its denaturation [86,87].

## 5. Strategies to Suppress Non-Specific Binding of Antibodies in EIs

History of immobilization procedures and design of biosensors start in 1962 when Clark and Lyons, 1962, developed the first enzyme membrane sensor for the detection of glucose in blood [88]. From this point until now, several methodologies related to the immobilization of well-oriented antibodies or protein layers onto electrode surfaces have been reported in order to improve the sensitivity of a wide variety of EIs. Enhancement of sensitivity can be achieved suppressing NSB of proteins [89]. 

**Figure 6:**
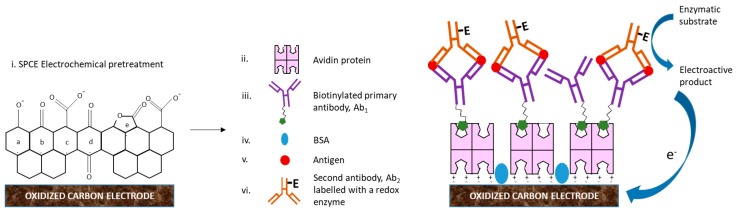
Fabrication steps of an EI based on neutravidin-coated SPCE surface in order to avoid NSB of immunoreagents. i). Pretreatment of a SPCE surface produce several functional groups: a. phenols, b. carbonyls, c. carboxyls, d. quinones and e. lactones. These functional groups in aqueous solutions with pH values equal to or above the physiological pH are deprotonated, which confer to the surface a negative net charge. ii). Neutravidin protein positively charged is adsorbed by electrostatic interaction on the negatively charged SPCE surface. iii). Biotinylated primary antibodies (Ab_1_) binds to neutravidin with high specificity and selectivity. iv). To suppress NSB of antigens (Ag) on neutravidin non-coated electrode surface regions, bovine serum albumin (BSA) is used as a blocking reagent. v). Biorecognition event between Ag and Ab_1_ for Ag detection. vi). A second antibody (Ab_2_) labelled with a redox enzyme (E). Redox enzyme oxidizes an enzymatic substrate to produce an electroactive product and electrons which reach the electrode surface originating an electrochemical signal response that can be correlated with the amount of antigen present in a sample.

In EIs design, one of the most studied and described techniques related to the high specific binding of oriented antibodies onto electrode surfaces is the avidin coating of surfaces [90] (Figure 6). Avidin is a protein originated from the egg white composed by four subunits called tetramers with the ability to bind one biotin per subunit or four biotin molecules per avidin molecule. The avidin-biotin system has the strongest non-covalent interaction between biomolecules known, with an incredible affinity constant of 10^15^ M^−1^ [91,92]. Also, it has been reported that analogous of avidin such as streptavidin and neutravidin have the same affinity for biotin molecules and contribute to the enhancing of analytical efficiency in EIs [90]. Neutravidin has the lowest NSB among all known binding proteins including streptavidin [93].

Another common strategy to avoid NSB in EIs is the use of block buffers such as superblock and superblock T20 in phosphate buffer solution (PBS) and Tris buffer solution (TBS), starting block and starting block T20 in PBS and TBS, casein and bovine serum albumin (BSA) in PBS and TBS. Among all these blocker reagents, BSA is the most frequently used [94,95]. The perfect blocking reagent must be able to occupy all the remaining NSB sites after the adsorption of the coated protein (Figure 6).

One widely used technique, due to its simplicity and good cost-efficiency for suppressing NSB of proteins on bare electrode surfaces, is the direct adsorption or passive adsorption of proteins also called physisorption in the form of a biologically active monolayer [96]. Protein physisorption is very often carried out through hydrophobic and electrostatic interactions (Figure 6). In general, it has been demonstrated that a strong physisorption of proteins occurs on bare surfaces of CPE and SPCE, as mentioned earlier [84,85]. 

To enhance the biocompatibility of the carbon electrode surface towards antibodies or proteins and minimize NSB, carbon electrode surfaces are functionalized. Functionalization is achieved by oxidation of the carbon surface with various oxidizing agents (O_2_, N_2_O, CO_2_, H_2_O) and using strong chemical solutions (KMnO_4_, HNO_3_, KClO_3_, H_2_SO_4_). Bare carbon surfaces of the SPCE for example, are pretreated electrochemically using a sulfuric acid solution and applying an oxidation potential [97,98]. Figure 6 summarizes in an EI design all the strategies mentioned before such as neutravidin-coated SPCE surfaces, blocking reagents and physisorption for suppressing NSB of proteins.

Electrode surfaces can be modified prior the protein adsorption to suppress NSB and assure the well oriented antibody immobilization in order to enhance sensitivity and selectivity of EIs. Chemical modification strategies include i) allotropic modification of carbon, e.g., carbon nanostructures, ii) modification by metal nanoparticles, e.g., Au/Ag nanoparticles and magnetic beads, iii) polymerization, e.g., polyethylene glycol (PEG), oligo(ethylene glycol) (oEG) and conducting polymers, iv) self-assembled monolayers (SAMs), v) diazonium salt surface chemistry and vi) sol-gel chemistry modification [5,6,89,99]. 

Carbon nanostructures also called carbon nanomaterials are mainly constituted by carbon nanotubes (CNTs): single-walled (SWCNTs) and multi-walled (MWCNTs) carbon nanotubes [100,101,102]; and graphene: graphene oxide (GO) and reduced graphene oxide (rGO) [103,104,105,106]. CNTs and graphene were discovery in 1991 and 2004 respectively and emerge as alternative strategies to suppress NSB and improve EIs sensitivity due to their excellent electrical and thermal conductivity, chemical stability and flexible mechanical properties [107,108]. 

Carbon nanomaterials have large specific electroactive surface area offering a lot of binding sites to bind specifically and selectively higher amounts of antibodies and proteins which means an enhancement in the signal amplification and hence improvement in the analytical response of EIs. Carbon nanomaterials increase biocompatibility of the carbon surface towards the antibodies and protein avoiding denaturation and loss of their binding activity during the adsorption process. Also, CNTs and graphene increase electron transfer rates of carbon electrode surfaces, the major concern that carbon sensor platforms have [109,110,111,112,113]. 

A disadvantage of CNTs is that their fabrication process is not completely controllable and parameters such as aggregation and uniformity of nanotubes need to be improved. Also, CNTs, as well as graphene, are water insoluble, which can be overcome by modifying their surfaces with hydrophilic functional groups in order to increase their solubility and suppress NSB of antibodies via a covalent bond, but for CNTs this is not effective and in the case of graphene the strategy is satisfactory [41,114,115]. 

**Figure 7:**
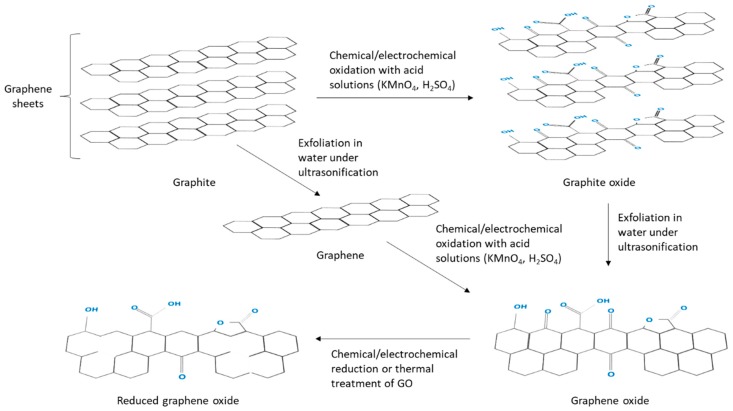
Preparation routes of graphene, GO and rGO carbon nanomaterials from graphite for application in EIs [124].

Graphene is a single two-dimensional (2-D) compact honeycomb hexagonal nanostructure of carbon atoms (Figure 7), and since its discovery, graphene has had greater interest than CNTs as supporting electrode material in EIs applications. Because graphene has a high density of defects on its surface where the highest electrochemical activity is concentrated [116], it also has high surface area, fast electron transfer rates, ease functionalization, high thermal conductivity, excellent mechanical strength and flexibility as well as biocompatibility and mass production [117,118,119]. However, reproducibility and stability of graphene electrode material needs to be improved [120]. Graphite can be oxidized to graphite oxide following the methodology proposed by Hummers et al. in 1958 and Kim et al. in 2010 [121,122], in order to obtain GO and rGO [123] (Figure 7).

GO has two relevant aspects: first, its fabrication cost is very low since the raw material is graphite and chemical methods used are cost-effective; and, second, GO is highly hydrophilic which means formation of stable aqueous-colloids for the assembly of more complex structures through simple and non-expensive methods. The principal difference between graphite oxide and GO is the number of layers. Since the graphite oxide system is composed of multilayers of functionalized graphene; GO comprises monolayers or few layers of functionalized graphene [124].

Recently, rGO has seen numerous applications in suppressing NSB of proteins in order to improve electrochemical response in EIs. Haque et al. in 2012 built an electrochemical reduced graphene oxide, ERGO-EI for the detection of mouse IgG using an ELISA sandwich format. They adsorb GO onto a pretreated indium tin oxide (ITO) electrode. The ITO surface electrode was modified with aminoethyl benzenediazonium (AEBD) salt with the amino terminal exposed to the solution. GO was attached covalently to the AEBD/ITO surface, and then was electrochemically reduced. After that, a layer of amphiphilic polymer poly (BMA-r-PEGMA-r-NAS) most commonly known as poly (BPN) was adsorbed onto the ERGO/AEBD/ITO platform. Once the electrochemical platform (poly (BPN)/ERGO/AEBD/ITO) was built, the immunocomplex sandwich was adsorbed via a covalent bond between the reactive end NAS (N-acryloxysuccinimide) of the poly (BPN) outer layer of the sensor platform and the amine groups present in the antibody. The sandwich immunocomplex comprises an anti-mouse IgG primary antibody, IgG used as antigen and an anti-mouse IgG secondary antibody labelled with HRP. Hydroquinone, HQ, was used as redox mediator and hydrogen peroxide (H_2_O_2_) was employed as enzyme substrate. Limit of detection (LOD) of this ERGO-EI was 100 fg/mL (700 aM) due to the enhanced electrocatalytic activity and the suppression of NSB of proteins [125].

Another important methodology to modify electrode surfaces in order to suppress NSB and enhance sensitivity and selectivity of EIs is the employment of metallic nanoparticles (MNPs). MNPs have been widely used as supporting electrode materials increasing electron transfer rates and surface-to-volume ratio to immobilize large amounts of primary antibodies and hence enhancing analytical response of the EIs devices. MNPs have also been employed in catalysis of electrochemical reactions, and biomolecule labelling [126,127,128]. 

A broad variety of MNPs in EIs applications include gold (Au) and silver (Ag) NPs. They have been used as electrode materials due to their excellent electrochemical (good electrical conductivity) properties, large surface-area compared to their volume, excellent optical properties, strong biomolecule adsorption, high stability, uniform particle size, and good biocompatibility towards antibody and protein in order to avoid denaturation and keep their binding capacity [129,130,131] Recently, easy, rapid and cost-effective NP preparation methods make these strategies more attractive for inclusion in EIs devices.

**Figure 8:**
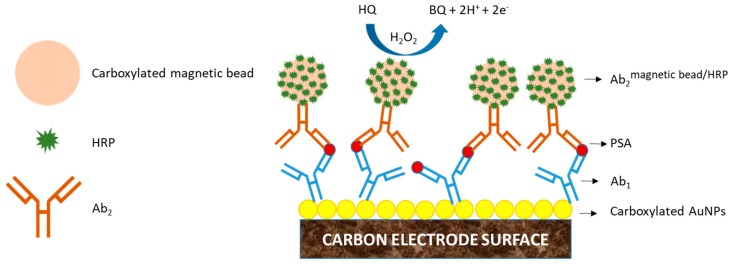
Diagram of the EI based on AuNPs electrode surfaces for the detection of PSA. Carboxylated AuNPs are electrostatically adsorbed onto PDDA modified carbon electrode surfaces. Ab_1_ were covalently adsorbed onto AuNPs electrode surfaces via amide bond formation. PSA antigen is captured by Ab_1_. To improve sensitivity and signal amplification of the EI, Ab_2_^magnetic bead/HRP^ complex was employed. HQ was used as redox mediator and H_2_O_2_ as enzyme substrate. Oxidation of HQ produce BQ, 2 mol of protons (H^+^) and 2 mol of e^−^ per mol of HQ involved in the electrochemical reaction. H_2_O_2_ is reduced to water and oxygen [132].

Mani et al. in 2009 developed an ultrasensitive EI using AuNPs film electrodes and magnetic beads labelled with HRP for signal amplification in the detection of prostate specific antigen (PSA). Glutathione-protected AuNPs (GSH-AuNPs) of 5 nm of size were deposited onto a cationic polydimethyldiallylammonium (PDDA) modified pyrolytic graphite and glassy carbon electrode. Negative charged GSH-AuNPs were electrostatically adsorbed using the layer-by-layer methodology. A proof of concept to measure functionality of the AuNPs electrode sensing platform was achieved attaching covalently HRP onto the carboxylate AuNPs electrode layer via amide bond formation. This PDDA/GSH-AuNPs/HRP platform was used for the electrochemical determination of hydrogen peroxide (H_2_O_2_). A limit of detection of 20 nM of H_2_O_2_ was found. Once the good functionality of the electrode sensor platform was proved, the electrochemical sandwich ELISA immunoassay for the detection of PSA was developed. Primary antibodies (Ab_1_) were covalently adsorbed onto AuNPs electrode surfaces via amide bond formation. A traditional sandwich ELISA assay was performed using a secondary antibody (Ab_2_) labelled with HRP (Ab_2_^HRP^). The LOD was found to be 1 ng/mL. Also, a signal amplification method using an Ab_2_ labelled with magnetic beads which are labelled with HRP enzymes (Ab_2_^magnetic bead/HRP^) was tested. The EI design for signal amplification is shown in Figure 8. To suppress NSB of labelled Ab_2_, 0.4% of casein and 0.05% of tween 20 in phosphate buffer solution (PBS) was used in order to achieve high sensitivity and low LOD. Hydroquinone (HQ) was used as redox mediator and H_2_O_2_ as enzyme substrate. Oxidation of HQ produce benzoquinone (BQ), 2 mol of protons (H^+^) and 2 mol of e^−^ per mol of HQ involved in the electrochemical reaction. H_2_O_2_ is reduced to water and oxygen. The LOD of PSA in this sensor platform array was 0.5 pg/mL, a signal enhancement of 2000 times in comparison with the value obtained from traditional Ab_2_-HRP labeled [132].

Silver NPs (AgNPs) present advantages such as lower cost and higher conductivity over other metallic NPs (copper, platinum, palladium, etc.). Despite the excellent properties of metallic NPs mentioned before and the widely used in EIs applications, it could be possible to achieve better values of analytical sensitivity. As an example, defects in the structure of rGO can be corrected using AgNPs which leads to an improvement in electrical conductivity by a factor of 346% [133]. 

**Figure 9:**
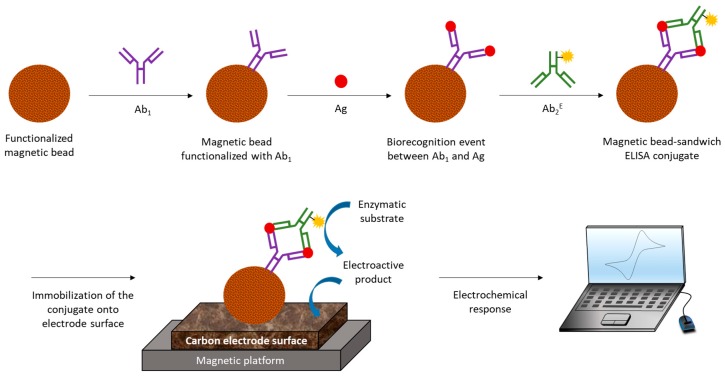
Schematic representation of typical EI fabrication steps using magnetic beads as surface material for immobilization of immunoreagents. The immunocomplex is composed of a primary antibody (Ab_1_), analyte of interest or antigen (Ag) and a secondary antibody (Ab_2_) labelled with a redox enzyme (E). The magnetic bead-sandwich ELISA conjugate is attached to the carbon electrode surface using a magnetic platform for electrochemical measurements in order to detect antigens of interest [134].

Magnetic beads have been broadly employed in the suppression of NSB of antibodies and enhancement in signal amplification in order to improve EI sensitivity. Many interesting characteristics of magnetic beads make them suitable for electrochemical sensing: high surface area, chemical and physical stability, low toxicity and biocompatibility [134]. Typically, magnetics beads are often used as substrates for the immobilization of antibodies and antigens in a sandwich ELISA format, and then they are brought onto carbon electrode surfaces with the help of a magnetic platform for the detection of the antigen of interest (Figure 9).

As an example, Zani et al. in 2010, employed protein G-functionalized magnetic beads solution to capture primary antibodies (Ab_1_) and carry out a sandwich ELISA format. The sandwich immunocomplex is composed of an Ab_1_, PSA antigen (Ag) and a secondary antibody (Ab_2_). A third antibody labelled with enzyme alkaline phosphatase (Ab_3_^E^) was added as a tracer in order to evaluate the immunocomplex formation. 1-naphthyl-phosphate was used as enzyme substrate. To avoid NSB between immunoassay reagents and the magnetic beads surface that remains without Ab1 functionalization, non-specific Ab_1_s were used as blocking reagent which were not able to bind with PSA. Then the magnetic bead/protein G/Ab_1_-Ag-Ab_2_-Ab_3_^E^ conjugate was brought to the carbon electrode surface with the help of a magnetic platform for separation and differential pulse voltammetry (DPV) determination of PSA. Limit of detection (LOD) was found to be 1.4 ng/mL for the PSA [135].

Functionalized water-soluble polymers such as polyethylene glycol (PEG) and PEG related materials, e.g., oligo(ethylene glycol) (oEG), are a good strategy as antifouling treatments of electrode surfaces preserving protein function and preventing NSB [136].

In electrochemistry, specifically in sensor applications, PEG grafting is a very useful tool to amplify the electron transfer. Hotchen et al. in 2015. investigated the effects of a PEG grafted glassy carbon electrode (GCE) and boron doped diamond electrode surface for polymerization degrees of n = 4.5 to 9.1 (PEG 200 to PEG400) in the “amplification” of the electron transfer rate [137]. 

Adsorption of redox proteins onto PEG electrografted modified GCE surfaces has been reported by Wen et al. in 2009. Hemoglobin, Hb, was immobilized onto modified glassy carbon with a substrate of PEG grafted-MWCNTs, synthesized through covalent attachment of MWCNTs with PEG chains functionalized with hydroxyl groups. Adsorption of Hb onto the PEG grafted-MWCNT GCE was determined using impedance spectroscopy and cyclic voltammetry. The novel composite exhibited excellent hydrophilicity and biocompatibility retaining a near-native conformation of the adsorbed hemoglobin as UV-vis spectroscopy confirms. Also, electrochemical properties of Hb on the PEG grafted-MWCNT modified GCE was carried out using cyclic voltammetry at a scan rate of 0.08 V/s, showing quasi reversible redox peaks centered at −0.34 V (vs. saturated calomel electrode) corresponding to the typical peaks of Hb Fe(III)/Fe(II) in PBS pH 7.0 [138]. 

Triethylene glycol (TEG), an oEG material was successfully used by Ren et al. in 2015 in the determination of dopamine (DA) in the presence of ascorbic acid (AA). Electrografted deposition of a film of TEG onto GCE showed selectivity for neutral species while ionic species, independently of their charge, were blocked by the film; this very interesting selectivity property for uncharged species allowed the determination of DA in the presence of AA using differential pulse voltammetry [139]. The backfilling approach is another very useful technique, instead of PEG and oEG grafting, wherein the spaces present in the long PEG chain are filled with shorter PEG chains serving as a protective layer to backfill the surface and suppress NSB [140]. Lokanathan et al. in 2011 used the backfilling approach for the self-assembled monolayer (SAM) of a mix of PEG thiol filled with oEG terminated alkane thiol molecules resulting in underbrush formation, causing a better protein resistance and less desorption of PEG compared to alkane thiol [141]. 

In the last two decades, conducting polymers have gained great interest in the improvement of sensitivity in EIs, due to their large surface area, electrical conducting properties over all its backbone thanks to the presence of an extended pi-orbital system, faster electron transfer rates, high sensitivity, specificity, structure stability and biocompatibility. The chemical structure of conducting polymers can be modified to regulate electrical and mechanical polymer properties according to the EIs application. The principal advantages of conducting polymers in the development of EIs over other available methodologies are the improvement of sensitivity and faster response time. Also, conducting polymers can be electrochemically-deposited in aqueous solutions at neutral pH onto a wide variety of electrode surfaces prior immobilization of immunoreagents. Other interesting characteristics of conducting polymers are their easy and cost-effective fabrication.

Several conducting polymers have been studied and include poly(acetylene)s, poly(pyrrole)s, poly(thiophene)s, poly(terthiophene)s, poly(aniline)s, poly(fluorine)s, poly(3-alkylthiophene)s, polytetrathiafulvalenes, polynapthalenes, poly(p-phenylene sulfide), poly(para-phenylene vinylene)s and others. Among all these, the two most studied conducting polymers employed as biorecognition elements for the immobilization of antibodies and proteins in general are polypyrrole (PPy) and polyaniline (PANI) [142,143].

**Figure 10:**
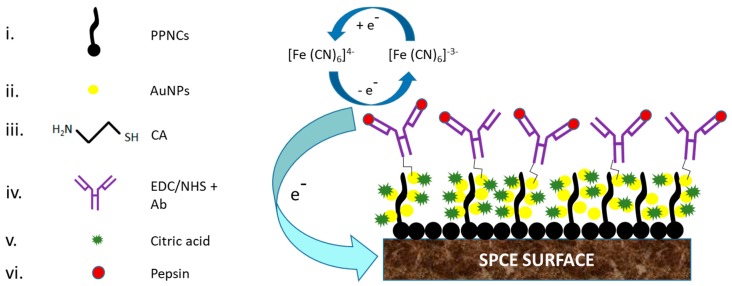
Fabrication methodology steps of an EI using AuNPs/PPNCs composites for carbon electrode modification in the detection of salivary pepsin. i). prenucleation and electropolymerization of pyrrole onto SPCE to produce PPNCs modified SPCE. ii). Electrochemical attachment of AuNPs on micelles PPNCs surface. iii). Cysteamine (CA) was conjugated to AuNPs via S-metal bond formation. CA was used as a spacer and linker between AuNPs and antibodies. iv). Antibody immobilization on CA/AuNPs/PPNCs/SPCE surface via amide bond formation using EDC/NHS conjugation mechanism. v). Citric acid was used as a blocker reagent, to avoid NSB of antigens on possible free binding sites that can be remaining in the AuNPs surface. vi). Detection of pepsin antigen in saliva using the [Fe (CN) _6_]^3−/4−^ redox probe. LOD of this sensor platform was found to be 2.2 ng/mL pepsin [144].

Lee et al. in 2018 developed an EI based on gold nanoparticles/polypyrrole nanocorals composite to modify an SPCE surface for the detection of salivary pepsin. Polypyrrole nanocorals (PPNCs) and gold nanoparticles (AuNPs) were used to suppress NSB of antibodies and enhance signal amplification. Different concentrations of pepsin (0, 6.25, 12.5, 25, 50 and 100 ng/mL) were added to the Ab/CA/AuNPs/PPNCs/SPCE EI platform (Figure 10). LOD of this sensor platform was found to be 2.2 ng/mL pepsin [144]. 

Self-assembled monolayers (SAMs), have gained great interest in EI design due to their capacity to attach well-oriented antibodies on electrode surfaces. Also, SAMs confer stability to the EI arrangement and a biocompatible substrate in order to preserve activity of subsequent attached biomolecules. Metal electrode surfaces are the most suitable materials for the use of SAMs. A broad variety of SAMs exists, but the most commonly used are based-on thiol groups present in the chemical structure of alkanethiols or in general polyelectrolytes. SAMs chemically modify the electrode surface, a process called chemisorption, through the formation of a metal-S bond between the metal electrode surface and the thiol group. 

**Figure 11:**
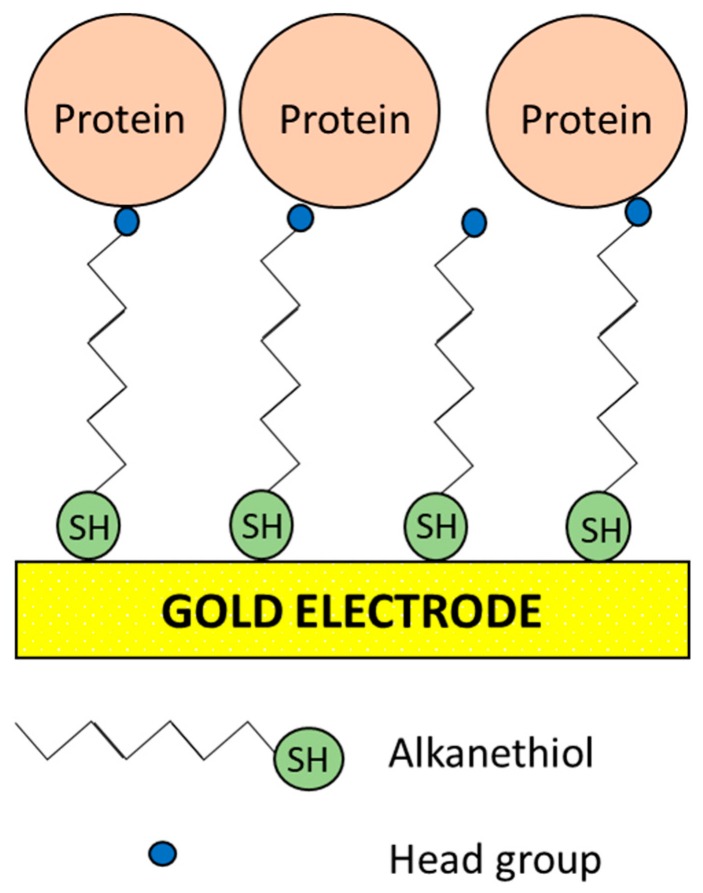
General scheme of the principal components of a SAM based on thiol groups. In the case of alkanethiol, the alkane aliphatic chain acts as a spacer arm in order to increase the degrees of freedom of the SAM and, in general, of the supramolecular architecture. Thiol tends to form a metal-S bond with the electrode surface leaving the head group exposed [85,146].

Another important feature of SAMs is their easy formation process, since it is only necessary to immerse the electrode in a solution of the chemical compound (polyelectrolyte) for an optimized period of time and wait for SAM formation. Generally, an alkanethiol molecule is composed of a head group, a spacer group and a terminal sulfur group also known as thiol (Figure 11). As we mentioned before, the thiol group tends to form a metal-S bond, attaching the alkanethiol to the electrode surface, while the head group remains exposed. An advantage of this head group is that it can be exchanged for molecules of interest to customize the physicochemical composition of the surface; for example, designing a hydrophobic surface by changing the head group with a methyl head group. An alkane aliphatic chain can act as a spacer arm in order to increase the degree of freedom of the SAM and, in general, of the supramolecular architecture [145,146,147].

One of the most relevant studies of SAMs applied in the construction of EIs platforms was developed by Corn et al. in 1995. A monolayer of biotinylated Poly (L-lysine), PL was used to control the NSB of avidin onto electrode surfaces. The functionalized PL with biotin (PL-Btn) was electrostatically adsorbed onto a gold surface previously modified through a covalent binding with a SAM of the polyelectrolyte 11-mercaptoundecanoic acid (MUA). The biotin molecules are exposed to the solution serving as a binding site available to avidin. The unwanted spaces that remain between avidin molecules after adsorption are blocked using BSA [63].

Delamar et al. in 1992 was the pioneer in the use of the diazonium salt chemistry which is a very promising methodology in the modification of electrode surfaces and is based on the electrochemical reduction of diazonium salts, producing a very compact and noncorrosive covalent attachment of aryl groups on the surface, allowing the immobilization of a wide variety of molecules [148]. Reduction of diazonium salts is carried out on different type of materials including carbon, semiconductor, metal, polymer and insulator surfaces. There are three unique characteristics of diazonium salt chemistry. The first one is the formation of a covalent bond between the substrate and the aryl groups [149]. The second one is the formation of a non-homogeneous surface topography and thickness of the aryl layers in the range of 2–6 nm [150]. The last feature is the formation of azo bonds within the first layer at the substrate-aryl layer interface and between the aryl multilayers [151]. For a detail revision of diazonium salt chemistry we invite you to consult the following references [152,153].

Qi et al. in 2012, developed a highly sensitive EI for the simultaneous detection of two important tumor markers, carcinoembryonic antigen (CEA) and α-fetoprotein (AFP) using a horseradish peroxidase (HRP)-labeled antibody as a signal antibody. Prior to the adsorption of the sandwich immune-complex, the electrode surface of an array of six SPCEs was electrografted with aminophenyl group by reduction of in situ generated aminophenyl diazonium cation generated from *p*-phenylenediamine. Diazonium salt solution containing NaNO_2_ and HCl was deposited onto the electrode surface and then electrochemically grafted using cyclic voltammetry in a potential range from 0.2 to −0.6 V with a scan rate of 50 mV/s for three cycles. LOD of the EI was found to be 0.03 ng/mL for CEA and 0.05 ng/mL for AFP (S/N = 3) [154].

Gam-Derouich et al. in 2012 developed a strategy for the preparation of molecularly imprinted (MIPs) sensing surfaces based on gold electrodes. Diazonium salt BF_4_^−^, ^+^N_2_–C_6_H_4_–CO–C_6_H_5_ was used as photoinitiator and then grafted by photopolymerization onto the gold surface using chronoamperometry and setting the potential at −700 mV. After this, MIP grafts for dopamine were prepared by surface-initiated photopolymerization (SIPP) using methacrylic acid (MAA) as the functional monomer (F) and ethylene glycol dimethacrylate (EGDMA) as the cross-linker monomer (R). Specificity and selectivity of the gold-grafted MIP (Au-MIP) electrodes toward dopamine were tested by square wave voltammetry (SWV) with a LOD of 0.9 nmol/L [155].

Finally, sol-gel chemistry is an alternative strategy to organic polymers and SAMs due to its greater compatibility with silicate glass matrices and adsorbed biomolecules. Advantages of biosensors based on sol–gel technology include their relative porosity, chemical inertness, simplicity of preparation, low temperature encapsulation, high sensitivity and their ability to retain the biological activity for a longer period of time. Moreover, the hydrophilic, porous and positively charged alumina sol–gel matrix provides a friendly microenvironment for the target molecules [156]. Sol-gel chemistry was used for the first time in the development of an electrochemical immunosensor by Wang et al. in 1998. An amperometric sensor was fabricated in one-step combining sol-gel and screen-printed technologies for the detection of rabbit immunoglobulin G (RIgG). Secondary antibody was labeled with alkaline phosphatase (AP), naphthyl phosphate as substrate, and amperometric detection at +400 mV (vs Ag/AgCl). Results showed a LOD of 5 ng/mL of RIgG [157].

For a detail revision of sol-gel chemistry we invite you to consult the following references [158,159,160].

## 6. Conclusions and Perspectives

The development of EIs that can be applied, for example, in the clinical diagnosis of diseases such as cancer and heart issues have been widely studied throughout these years. It is notorious the interest to create new miniaturized EIs platforms that meet the following requirements:

- Portability for point of care testing applications
- Low cost disposable EIs
- High sensitivity and specificity to the target molecule
- Rapid clinical analysis
- Low sample and reagent consumption
- Easy to use

These goals can be achieved by taking into account a very large variety of phenomena that occur simultaneously in the protein-electrode interface when the adsorption of the protein happens. Physicochemical properties of the protein and the surface play an important role when the method to modify electrode surfaces has to be chosen. Different techniques to avoid NSB of proteins have been reported, but in general, the most fundamental feature at the moment to build a sensor platform is to consider the final application. A well-defined and characterized supramolecular architecture of an EIs for the detection of a particular biomolecule such as a cancer/cardiovascular biomarker, can be adapted to detect other type of biomarkers, just by changing the biorecognition element against the target molecule. The major concern in the building of EIs regarding sensitivity is a poor signal response, which can be due to: the denaturation or loss of affinity/specificity of antibodies and proteins when they are adsorbed onto the electrode surface; an incorrect orientation of antibodies in its adsorbed state which leads to an increment of the steric hindrance and cross-reactivity from antibodies to other non-desired molecules present in the sample instead of the antigen of interest. 

Future studies in the design of new EIs platforms are based on the use of all these NSB-approaches discussed before in order to improve the analytical performance of EIs and apply them to build electrochemical-POCT version devices such as lateral flow immunochromatography and microfluidics devices giving place to the microfluidic electrochemical immunosensors (MEIs).

## Supplementary Materials

The video abstract are available online at https://drive.google.com/file/d/12MXggOawImrLmpi9Nq3xetu9UrkLG3Cs/view?usp=sharing.
